# Cytokinesis is blocked in mammalian cells transfected with *Chlamydia trachomatis *gene *CT223*

**DOI:** 10.1186/1471-2180-9-2

**Published:** 2009-01-05

**Authors:** Damir T Alzhanov, Sara K Weeks, Jeffrey R Burnett, Daniel D Rockey

**Affiliations:** 1 Department of Microbiology, Oregon State University, Corvallis, OR, 97331, USA; 2 Department of Biomedical Sciences, College of Veterinary Medicine, Oregon State University, Corvallis, OR, 97331, USA; 3 Department of Biochemistry and Molecular Biology, Oregon Health & Science University, Portland, OR, 97239-3098, USA

## Abstract

**Background:**

The chlamydiae alter many aspects of host cell biology, including the division process, but the molecular biology of these alterations remains poorly characterized. Chlamydial inclusion membrane proteins (Incs) are likely candidates for direct interactions with host cell cytosolic proteins, as they are secreted to the inclusion membrane and exposed to the cytosol. The *inc *gene *CT223 *is one of a sequential set of orfs that encode or are predicted to encode Inc proteins. CT223p is localized to the inclusion membrane in all tested *C. trachomatis *serovars.

**Results:**

A plasmid transfection approach was used to examine the function of the product of *CT223 *and other Inc proteins within uninfected mammalian cells. Fluorescence microscopy was used to demonstrate that *CT223*, and, to a lesser extent, adjacent *inc *genes, are capable of blocking host cell cytokinesis and facilitating centromere supranumeracy defects seen by others in chlamydiae-infected cells. Both phenotypes were associated with transfection of plasmids encoding the carboxy-terminal tail of CT223p, a region of the protein that is likely exposed to the cytosol in infected cells.

**Conclusion:**

These studies suggest that certain Inc proteins block cytokinesis in *C. trachomatis*-infected cells. These results are consistent with the work of others showing chlamydial inhibition of host cell cytokinesis.

## Background

Chlamydiae are obligate intracellular bacteria that replicate in a cytoplasmic vacuole (the inclusion) within host cells [1,2]. All *Chlamydia spp*. are significant pathogens, and infections occur in a wide variety of animal species. *Chlamydia trachomatis *infections lead to serious mucosal diseases of humans including blinding trachoma [3] and diseases of the genital tract [4]. The study of chlamydial host-pathogen relationships is complicated by the lack of a genetic system to manipulate the chlamydial genome, and thus, alternate approaches must be used to understand chlamydial virulence properties. One approach that has been particularly useful in these studies is the use of surrogate genetic systems including yeast, mammalian cells, and other bacterial species [5-10].

Inhibition of the host cell cycle by chlamydiae was demonstrated by early researchers [11,12] and was expanded upon recently by Greene and Zhong [13]. Other recent investigations have demonstrated that chlamydial infection alters the cell cycle in a variety of ways, leading to centrosomal defects [14] and slowing of host cell division [15]. The molecular mechanisms leading to these changes are poorly understood. Recent studies have suggested a possible role of chlamydiae in cancers of different infected tissues [16-18] and, thus, the role of chlamydiae in alterations of cell cycle biology are of significance.

The different chlamydial species each produce a set of proteins, termed Incs, that are localized to the chlamydial inclusion membrane and exposed to the cytosol of the host cell [19]. Each sequenced chlamydial genome encodes over 40 candidate Incs, and there are both conserved and species-specific Incs among the different chlamydiae. The demonstrated function of a limited number of Inc proteins is known [9,20-23], but most are poorly characterized.

*Chlamydia trachomatis *encodes a species-specific set of Incs within orfs *CT223*-*CT229*. *CT224 *and *CT225 *have no clear homologs in any other chlamydiae, while *CT223*, and *CT226–CT229 *have homologs only in *C. muridarum*, a closely related chlamydial species [24]. The localization to the inclusion membrane of the products of *CT223*, *CT225*, *CT226*, and *CT229 *was confirmed via fluorescence microscopy [25]. Transcription of *CT228 *and *CT229 *is initiated very early following infection of cells [26] and, therefore, the encoded proteins are hypothesized to be essential to early inclusion development. Recent work by Rzomp *et al. *demonstrated that CT229p associated with Rab4 in a two-hybrid assay and in mammalian cells [20], but the function of any of the proteins encoded by the other orfs in this group is not known. To address possible functions of candidate *C. trachomatis *Incs, we used a plasmid transfection system to introduce genes encoding different Incs into mammalian cells, and then characterized any resulting phenotypes with fluorescence microscopy. These investigations demonstrated that transfection with plasmids expressing *CT223*, and to a lesser extent, *CT224 *and *CT225*, led to a block in host cell cytokinesis. Cells transfected with plasmids encoding CT223p led to an inhibition of cytokinesis that was similar to that seen in *C. trachomatis-*infected cells. The block was shown to be associated with the carboxy-terminal end of CT223p, the region of the protein hypothesized to be exposed to the host cell cytosol at the surface of the inclusion. Alleles of *CT223 *from different strains yielded similar inhibition of cytokinesis, consistent with the inhibitory effect on cytokinesis by all tested *C. trachomatis *serovars [13].

## Methods

### Chlamydial strains, DNA preparation, and host cell lines

Elementary bodies (EB) of *Chlamydia trachomatis *strains D/UW3, J/UW36, J9235, J(s)1980, J(s)6686 and LGV-434, *C. caviae *strain GPIC, and *C. muridarum *strain Nigg were used in infections and/or for preparation of genomic DNA samples that were used as PCR templates. Genomic DNA was prepared by boiling EB suspensions in a water bath for 10 minutes followed by removal of bacterial debris via centrifugation. HeLa or McCoy cells (ATCC) were cultured in Minimal Essential Medium supplemented with 10% fetal bovine serum and 10 μg/ml gentamicin and grown at 37°C in 5% CO_2_.

### Construction of plasmids and oligonucleotides used in transfection experiments

Chlamydial open reading frame (orf) designations will be in italics and will reflect their numerical assignment in the *C. trachomatis *genome sequence presented by Stephens *et al. *[27]. The single *C. muridarum *orf tested (*TC0495*) was as predicted using the sequence of Read *et al*. [28]. The encoded chlamydial protein will be shown in regular text and will be followed by a "p". The plasmid pcDNA4/HisMaxC (Invitrogen) was used for cloning and expression of intact or truncated coding regions of *C. trachomatis CT223*, *CT224*, *CT225*, *CT226*, *CT227*, *CT228*, *CT229*, *incA, incC*, *C. caviae incA, incB*, *incC*, and *Aequorea victoria gfp *genes. Plasmids were constructed that encoded the carboxy terminal 179 and 56 amino acids of CT223p (CT223/179p and CT223/56p, respectively), and the amino acids between positions 91 and 214 of CT223p (CT223/91p). pcDNA4/HisMaxC encodes a polyhistidine tag that was fused to the amino terminus of each recombinant polypeptide tested. Oligonucleotides were designed to include appropriate restriction sites for cloning (Table 1). PCR reactions were carried out using *Pfx *polymerase and chlamydial genomic DNA as template, and the entire coding sequence as predicted from the serovar D genome sequence was used to define the orfs. All constructs were confirmed by nucleotide sequence analysis.

**Table 1:** Oligonucleotides used for amplification of *inc *genes by PCR.

Name/Site	Sequence	Target Gene
DA71 *Eco*RI	agcaGAATTCttgagatctagaaaagaagc	*CT223 C. trachomatis*
DA97 *Kpn*I	agcaGGTACCaatggtgagtttagcagg	*CT223 C. trachomatis*
DA116 *Eco*RV	agcaGATATCctacacccgagagccattg	*CT223 C. trachomatis*
DA119 *Eco*RV	agcaGATATCctaattagccgttttcagatt	*CT223/179 C. trachomatis*
DA121 *Eco*RV	agcaGATATCctactcttctatctgctcttt	*CT223/91 C. trachomatis*
DA122 *Eco*RI	agcaGAATTCatggagcttaaagctttagag	*CT223/56 C. trachomatis*
DA76 *Bam*HI	agcaGGATCCttattttttacgacgtgc	*CT229 C. trachomatis*
DA99 *Kpn*I	agcaGGTACCaatgagctgttctaataa	*CT229 C. trachomatis*
DA98 B*am*HI	agcaGGATCCatgagtactactattgg	*CT228 C. trachomatis*
DA74 *Pst*I	agcaCTGCAGctaagaagcttggttgtc	*CT228 C. trachomatis*
DA 131 *Eco*RI	agcaGAATTCatgtcttatcttttttcc	*CT227 C. trachomatis*
DA 132 *Eco*RV	agcaGATATCtcatgagacacttatcac	*CT227 C. trachomatis*
DA 129 *EcoR*I	agcaGAATTCatgttggccttttttcga	*CT226 C. trachomatis*
DA130 *Eco*RV	agcaGATATCttatatcagactttccaa	*CT226 C. trachomatis*
DA127 *Eco*RI	agcaGAATTCatggtggctaacaactttatt	*CT225 C. trachomatis*
DA128 *Eco*RV	agcaGATATCttaatcccacccatgttt	*CT225 C. trachomatis*
DA125 *Eco*RI	agcaGAATTCatgagttttgttggaagt	*CT224 C. trachomatis*
DA126 *Xho*l	agcaCTCGAGctaatcattgggaaatga	*CT224 C. trachomatis*
DA34 *Eco*RI	agcaGAATTCatgacaacgcctactact	*incA C. trachomatis*
DA21 *Eco*RV	agcaGATATCctaggagctttttgtggg	*incA C. trachomatis*
DA22 *Eco*RI	agcaGAATTCggcaacgttatgacgtc	*incC C. trachomatis*
DA23 *Eco*RV	agcaGATATCttagcttacatatatttg	*incC C. trachomatis*
JL003 *Eco*RI	agcaGAATTCatgacagtatccacaaa	*incA C. caviae*
JL010 *Eco*RV	agcaGATATCacttaactatctttatc	*incA C. caviae*
JL007 *Eco*RI	agcaGAATTCatgtcaacaacaccatc	*incB C. caviae*
JL006 *Eco*RV	agcaGATATCttaagattctgtttgat	*incB C. caviae*
JL014 *Eco*RI	agcaGAATTCatgacctctgtaagaga	*incC C. caviae*
JL013 *Eco*RV	agcaGATATCtaaatgtccggtaggag	*incC C. caviae*
DA114 *Eco*RI	agcaGAATTCatggtgagcaagggcga	*GFP*
DA115 *Eco*RV	agcaGATATCctacttgtacagctccatg	*GFP*

The restriction sites built into oligonucleotides for cloning purposes are shown in capital letters.

### Antibodies, transfection experiments and immunofluorescence microscopy

Monoclonal antibody recognizing chlamydial lipopolysaccharide was a gift from Harlan Caldwell of the Rocky Mountain laboratories, Hamilton, MT. Monoclonal antibody A57B9 (anti-HSP60) recognizes a genus common epitope on chlamydial HSP60 protein [25]. Monoclonal antibodies used in the analysis of CT223p localization in *C. trachomatis*-infected HeLa or McCoy cells were produced and used as previously described [25]. Rabbit polyclonal anti-CT223p antisera was generated against the peptide sequence NH_3_-NGINDLSPAPEAKKTGSGL and were produced commercially (Proteintech, Chicago, IL). For these experiments, cells were infected with chlamydiae and incubated for time periods indicated in the figure legends. Cells were then fixed with 100% methanol and used for immunofluorescence.

Transfection of plasmids into HeLa or McCoy cells grown on sterile glass coverslips was conducted using Lipofectamine 2000 (Gibco) according to the manufacturer's instructions. Transfected cells were incubated for 36 hours and then fixed with methanol. The efficiency of transfection was determined by labeling with monoclonal anti-6-His antibody (Clontech) and secondary FITC or TRITC fluorescent antibodies (Southern Biotechnology Associates) to detect the product of the transgene. Monoclonal anti-γ-tubulin antibodies (Sigma) were used to detect centrosomes. Cells expressing *gfp *were analyzed without labeling. Coverslips were examined under 1000× magnification using a Leica fluorescence microscope and images were collected using the SPOT digital camera system (Diagnostic Instruments Inc., Sterling Heights, MI). The rates of cells with a polynuclear phenotype were determined by counting transfected cells with two or more nuclei among the total population of transfected cells.

### Statistical analysis

The number of transfected cells having a polynuclear phenotype was evaluated in at least three independent experiments for each plasmid construct tested. A total of at least 500 individual transfected cells were counted for each tested plasmid construct. Standard deviations were calculated for each individual plasmid construct examined and the significance of differences between means was evaluated using both the Student's t-test and the Kruskall-Wallis test, as calculated using the Instat software program (GraphPad Software, San Diego, CA).

## Results

### Examination of the association between infection and a reduced rate of cytokinesis

**Figure 1:**
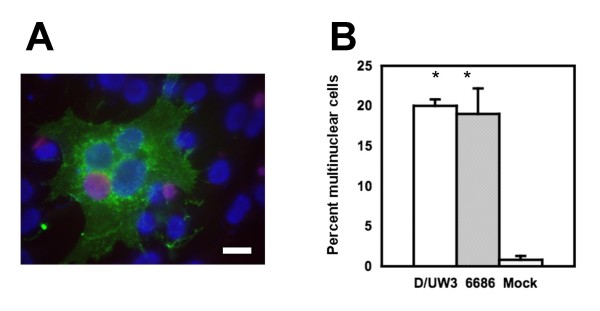
**Confirmation of the polynuclear phenotype in cells infected with different *C. trachomatis *strains**. Panel A: Fluorescence micrograph of *C. trachomatis *strain LGV-434 inclusion (anti-LPS, red) within a GFP-positive cell (green), showing three nuclei (blue). The scale bar indicates 10 microns. Panel B: The percentage of polynuclear cells 30 h after infection of HeLa cells with different *C. trachomatis *at an MOI of 3. Strains D/UW3 and J(s)6686 are shown, along with mock-infected cells. Statistical significance is indicated with the asterisk above the individual treatment groups, as compared to mock-transfected cells (Student's t-test, p < 0.001). Similar levels of significance were observed in a Kruskall-Wallis test (not shown).

The block in cytokinesis identified by Greene and Zhong [13] was tested using laboratory prototype strains (LGV-434 and D/UW3) and a recent clinical isolate {J(s)6686}. In experiments with multiplicities of infection of approximately 3, an increase in the polynuclear phenotype was verified both qualitatively (Fig. 1A) and quantitatively (Fig. 1B). These results are consistent with their data using laboratory strains and confirm that *C. trachomatis *infection blocks or slows cytokinesis in infected cells.

### Distribution of CT223p at the inclusion membrane varies in different *C. trachomatis *strains

**Figure 2:**
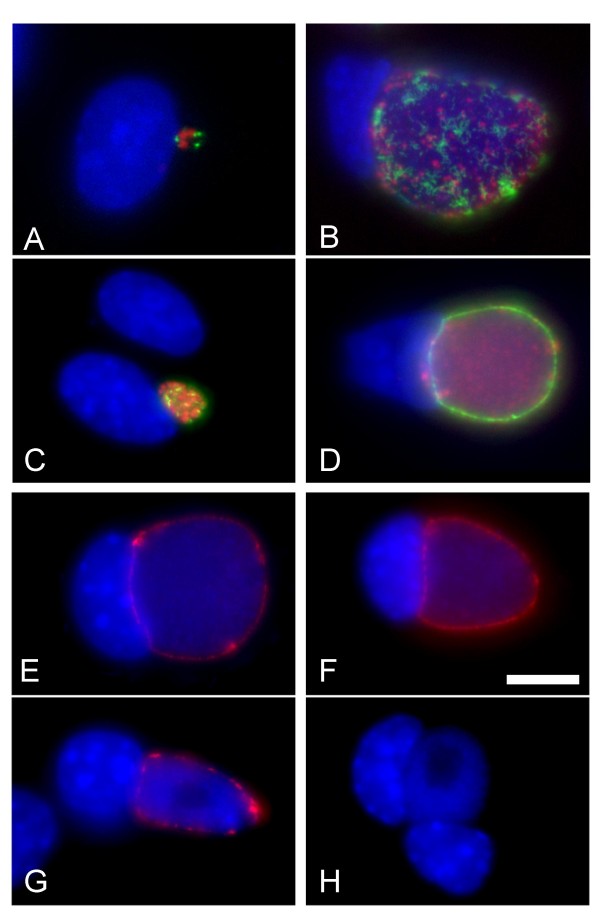
**Expression of *CT223 *at different times post infection and differential reactivity with specific antibodies**. DNA in all panels is labeled with DAPI (blue) and the bar in panel F represents 10 microns in each image. Cells were infected at an MOI of approximately 0.2 and fixed with 100% methanol prior to antibody labeling. Panels A-D: Fluorescent microscopy of McCoy cells infected with either strain LGV-434 (A, B) or J/UW36 (C, D). Cells were fixed at different times p.i. (A: 12 h, C: 18 h, B, D: 38 h). In panels A-D, cells were labeled with monoclonal anti-CT223p antibody (green) and anti-HSP60 (red). Note that labeling of CT223p is patchy in each strain at the early times points p.i. (A, C) but the labeling is distinct between strains at 38 h p.i. (B, D). Panels E-H: Cells were infected with strain J/UW36 (E, F) or J(s)1980 (G, H) and fixed 30 hours p.i. Cells were then labeled with either polyclonal anti-CT223p antisera (E, G) or monoclonal anti-CT223p antibody (F, H), both of which are labeled red. Note that CT223p is labeled by the polyclonal antisera in each strain, while the monoclonal anti-CT223p does not label the protein in strain J(s)1980.

CT223p is localized to the inclusion membrane in cells infected by *C. trachomatis *at time points after 8 hours post infection (p.i.). Consistent with our previous work [25], patches of CT223p protein are readily detectable at time points 12 h p.i. and later (Fig. 2A–D). The localization of CT223p is different in cells infected by representatives of different *C. trachomatis *serovars. In cells fixed at early and middle time points p.i., the labeling in cells infected by different serovars is similar and is manifested as dash-like or patchy localization of protein at the inclusion surface (Fig. 2A, C). At late time points however, a difference becomes apparent, as the labeling CT223p of a serovar J isolate (Fig. 2D) becomes more diffuse than in isolates of serovar L2 (Fig. 2B) and serovar D (not shown). These differences in labeling are independent of cell type (either McCoy or HeLa) or fixative (paraformaldehyde or methanol).

We have shown that CT223p in certain strains – including J(s)1980 and J(s)6686 – is not recognized in fluorescent microscopic analysis using two different anti-CT223p monoclonal antibodies [25,29] (Fig. 2F, H). However, peptide-specific polyclonal antibodies demonstrate that the protein is produced in all tested strains (Fig. 2E, G).

### Delivery of full length and carboxy-terminal *C. trachomatis *CT223p to the host cell cytosol alters host cell phenotype

**Figure 3:**
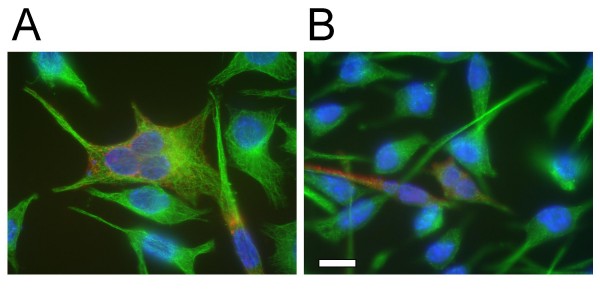
**Cytosolic production of CT223p and CT223/179p from *C. trachomatis *serovar D/UW3 leads to a multinuclear phenotype within mammalian cells**. The vector pcDNA4/HisMaxC was used in each construct. Full length CT223p (panel A) and CT223/179p (panel B) were produced within cells following transfection of pcDNA4-based plasmids. Each was detected with anti-6 × His monoclonal antibodies (red). Microtubules were detected by labeling with specific anti-tubulin antibodies (green). The nuclei are labeled with DAPI (blue). Panel A; McCoy cell transfected with pcDNA4/HisMaxC encoding CT223p. Three nuclei are localized inside of a single cell expressing *CT223*. Panel B; McCoy cells transfected with pcDNA4/HisMaxC encoding carboxy-terminal CT223/179p. The scale bar in B indicates 10 microns for each panel.

**Figure 4:**
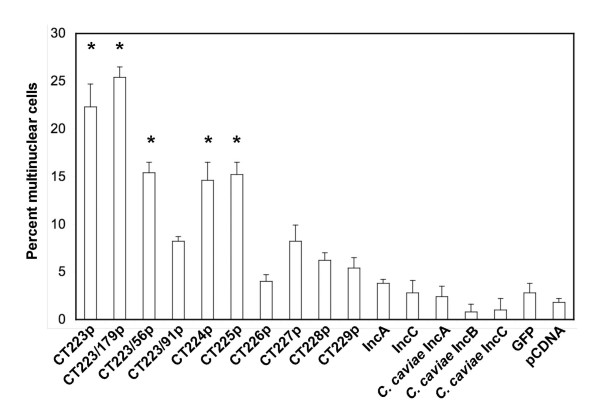
**Quantification of multinuclear cells following expression of different *inc *genes in McCoy cells**. This graph represents percentage of polynuclear cells among McCoy cells following transfection of pcDNA4/HisMaxC-based plasmids encoding different Inc proteins. Unless indicated, the sequences were derived from the published *C. trachomatis *D/UW3 genome sequence. Statistical significance is indicated with the asterisk above the individual treatment groups, as compared to pCDNA-transfected cells (Student's t-test, p < 0.01).

**Figure 5:**
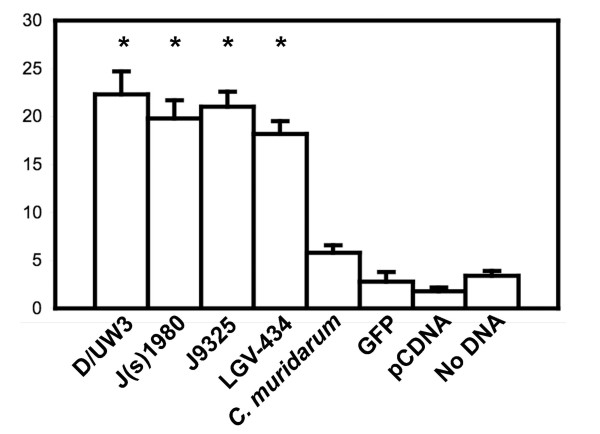
**Quantification of multinuclear cells following expression in McCoy cells of *CT223 *alleles from different *C. trachomatis *strains**. Statistical significance is indicated with the asterisk above the individual treatment groups, as compared to pcDNA-transfected cells (Student's t-test, p < 0.01).

Plasmids encoding CT223p from several *C. trachomatis *strains were transfected into both McCoy or HeLa cells and the effect on cellular cytokinesis was observed using fluorescent microscopy. Transfection with each of these plasmids led to a high proportion of multinucleate cells 30 hours post transfection (Fig. 3A). A similar phenotype was observed when cells were transfected with plasmids encoding the carboxy-terminal tail of CT223p (Fig. 3B). The average number of polynuclear cells following expression of a *CT223 *transgene was approximately 20%, regardless of the isolate from which the gene was amplified (Figs. 4 and 5). In contrast, cells transfected with a plasmid encoding GFP, or cells transfected with an empty vector (mock transfected) as control, all had levels of polynuclear cells of approximately 2–4%.

The multinuclear phenotype was manifested by the carboxy-terminal 179 amino acids of CT223p (Fig. 4). A reduced but still significant level of multinuclear cells were identified in cells transfected with a plasmid encoding only the carboxy-terminal 56 amino acids of CT223p, but, transfection of a plasmid encoding an internal fragment of CT223p (CT223/CT91p) did not lead to a significant level of multinuclear cells. These data suggest that the domain of the protein responsible for blocking cytokinesis was present in the carboxy-terminal 56 amino acids.

### Cytosolic expression of other *incs*

The orf encoding CT223p is within a likely operon encoding known and candidate *inc *genes *CT223–CT227*, and is adjacent to a very early operon containing two *inc *genes (*CT228 *and *CT229*). We tested each orf in these operons for an association with a polynuclear phenotype. Each orf was expressed in transfected cells and there was no apparent difference in expression level, based on fluorescence microscopy of transfected cells (not shown). Orfs *CT224 *and *CT225 *also were associated with a reduced but still statistically significant percentage of polynuclear cells in a transfected population (Fig. 4). None of the other tested orfs were associated with an increased number of polynuclear cells.

The same approach was used for testing the effects on cell cytokinesis of other Inc proteins. HeLa or McCoy cells transfected with plasmids encoding each protein from *C. trachomatis incA *and *incC*, and *C. caviae incA*, *incB *and *incC *were compared with cells expressing full length and truncated *CT223*. None of these plasmids led to an increase in polynuclear cells relative to controls (Fig. 4).

The *CT223 *coding sequence from different *C. trachomatis *strains encode proteins with up to 5% difference in amino acid sequence (22). We therefore tested plasmids encoding CT223p from strains with known amino acid sequence differences for their ability to block cytokinesis. Transfection of plasmids encoding each CT223p sequence was associated with an increase in multinucleate cells (Fig. 5). In contrast, transfection of a plasmid expressing *C. muridarum TC0495*, which is a syntenous, apparent *CT223 *homolog (less than 30% predicted amino acid sequence identity), did not lead to an increase in the number of multinucleate cells relative to controls (Fig. 5).

### Cells producing CT223p and CT223/179p have increased numbers of centrosomes

**Figure 6:**
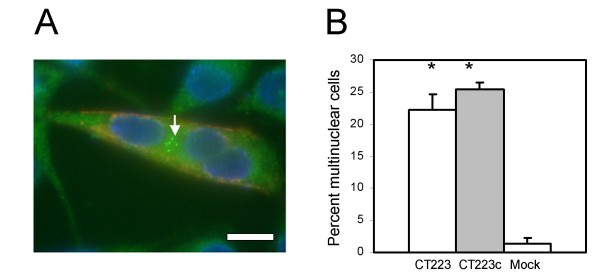
**Centrosome supranumeracy in cells transfected with plasmids encoding *C. trachomatis *serovar D CT223p and CT223/179p**. The vector pcDNA4/HisMaxC was used in each construct. The proteins CT223p and CT223/179p were detected with anti-6 × His monoclonal antibody and are labeled in red. Structures of γ-tubulin were detected by labeling with anti γ-tubulin antibodies and are stained in green. The nuclei are labeled with DAPI (blue). Panel A; McCoy cell transfected with pcDNA4/HisMaxC encoding CT223p. Three nuclei are localized inside of a single cell expressing CT223. Multiple centrosomes are shown with an arrow. The scale bar indicates 10 microns. Panel B; The percentage of cells with multiple centrosomes among cells transfected with plasmids encoding CT223p or CT223/179p (CT223c), or cells transfected with the pcDNA4/HisMaxC vector only (Mock). The vertical axis indicates the percent of cells that had two or more centrosomes. At least 500 cells were tested for each construct. The proportions of cells containing 2 or more centrosomes were significantly different than the mock-transfected cells for both the full length and truncated *CT223 *sequences. Statistical significance is indicated with the asterisk above the individual treatment groups, as compared to mock-transfected cells (Student's t-test, p < 0.001).

To further explore the multinuclear phenotype, cells expressing *CT223 *were labeled with antibodies specific against γ-tubulin. In contrast to α- and β-tubulin, which are structural components of microtubules, one of the functions of γ-tubulin is the nucleation of microtubule growth in all eukaryotes [30]. The microtubular organizing center, or centrosome, can therefore be identified with antibodies to γ-tubulin. We conducted transfection experiments with plasmids encoding both full length CT223p and the truncated CT223/179p molecule, and these cells also had statistically significant increases in the number of centrosomes, relative to control transfections (Fig. 6). These results are consistent with those of Grieshaber et al. [14], who demonstrated that there are centrosomal supranumeracy defects in *C. trachomatis*-infected cells.

## Discussion

CT223p is a chlamydial Inc protein that varies antigenically but is produced by all tested *C. trachomatis *isolates. The protein was detected in our analysis at 8 h p.i. (not shown) and was abundant on the inclusion membrane at all subsequent time points. This is consistent with the transcriptional profiling of Belland *et al. *[26], who demonstrate that the transcript for *CT223 *is first detected 8 h p.i. and remains actively transcribed for the rest of the developmental cycle. The gene is clustered with a set of orfs (*CT223–CT229*) encoding known or candidate inclusion membrane proteins that are only found in the *C. trachomatis *and *C. muridarum *genomes [24]. CT223p is localized as patches or short ribbon-like distribution in all strains examined prior to 30 h p.i. At later time points the protein is differently distributed in different strains, shown in this work in a comparison between a serovar J strain and a serovar L2 strain. Tested isolates of serovar D appear similarly to the serovar L2 strain (not shown).

The ability of *C. trachomatis *to affect the mammalian cell cycle has been documented by several authors. Greene and Zhong [13] established that infection by *C. trachomatis *affects host cell cytokinesis in a multiplicity of infection-dependent manner, results that were confirmed in our work (Fig. 1). Grieshaber *et al. *[14] demonstrated that chlamydial inclusions associate with the centrosome leading to increased numbers of centrosomes and chromosome segregation defects in infected cells. Molecular interactions between chlamydiae and host molecules important in cell division were explored by Balsara *et al*. [15] who showed that chlamydial infection leads to alterations in the abundance of cyclin-dependent kinases and to the cleavage of cyclin B1. However, any chlamydial proteins that might participate in the alteration of the host cell cycle have not been identified.

While it is possible that the observed multinuclear phenotype is a function of cellular fusion, as opposed to inhibition of cytokinesis, Greene and Zhong [13] discuss several lines of evidence that point to the latter possibility. This includes the lack of observed fusion intermediates, the presence of mitotic forms, and normal DNA synthesis in chlamydiae-infected host cells. These observations support the likelihood that cells are being blocked in a terminal state of division, as opposed to being stimulated to fusion with neighboring cells, following chlamydial infection.

CT223p was first examined as a candidate Inc protein because of the presence of an amino-terminal bi-lobed hydrophobic domain that is proposed to be a membrane anchor for Incs [25]. Like many Incs, CT223p also contains a long carboxy-terminal tail that is largely hydrophilic. It is likely that this carboxy-terminal region of the protein is responsible for direct interactions between Incs and proteins in the host cell cytosol, a property shown to be true for tested Inc proteins [7,21,22]. Transfection of cells with plasmids encoding only the carboxy-terminal 179 amino acids or (to a lesser extent) the 56 carboxy-terminal amino acids of CT223p led to increased accumulation of host cell nuclei within cells. We have sequence data for CT223p from several *C. trachomatis *isolates and, while there is sequence variation among strains, the carboxy-terminal third of the protein is highly conserved [[29]; data not shown].

Two other Inc proteins, CT224p and CT225p, also affected host cell cytokinesis, although the effect was less than that observed with CT223p. These proteins are encoded sequentially in the *C. trachomatis *genome and are unique to this species. However, the predicted protein sequences of these three proteins share very limited primary amino acid identity. In contrast, the protein product of *C. muridarum *orf *TC0495*, an apparent homolog of *CT223 *that is encoded in a syntenous operon [29] did not block cytokinesis in our assays. The degree of identity between CT223p and TC0495p is not high, and it is likely that key structures between the two proteins are not conserved in a way that allows the *C. muridarum *protein to affect cytokinesis in this assay. The degree of identity among CT223p, CT224p and CT225p is even lower, and, therefore, it is even less intuitive that these proteins would share a common phenotype when produced within mammalian cells. Therefore, the molecular mechanisms associated with the inhibition of cytokinesis observed in these studies remain unclear.

There are many possible steps in the complicated process of cell division that might be affected by the Incs that affect cytokinesis. The cell cycle is under control of a family of protein kinases known as Cyclin-dependent kinases (Cdks), which are under control of various regulatory proteins such as CAK and CKIs [31,32]. Some of these proteins are differently processed or differently abundant in chlamydiae-infected vs. uninfected cultured cells [15]. We hypothesize that CT223p and other Inc proteins directly or indirectly disrupt Cdk, cyclin, or possibly other protein functions and, thus, affect cell cycle control. We are currently using surrogate systems to identify possible host cell cycle-specific proteins that interact directly with CT223p at the inclusion membrane surface.

## Conclusion

Plasmid-based expression of the chlamydial inclusion membrane protein CT223p caused a reduction in mammalian cell cytokinesis in vitro. Other Inc proteins had a lesser effect on cytokinesis in this assay. These results support the conclusion that *Ct223 *expression by *C. trachomatis *and localization of the protein to the inclusion membrane is associated with the observed inhibition of host cell cytokinesis in *C. trachomatis*-infected host cells.

## Authors' contributions

DR is the senior investigator on this study and participated in the design and evaluation of all work. DA was the primary investigator who conducted or directed the experiments. DA also wrote the different drafts of the manuscript. JB was an undergraduate student researcher who contributed significantly to the molecular cloning involved in this work. SW was a research assistant who contributed to both the experimentation and organization of the data.
